# Treatment-induced mucositis: an old problem with new remedies.

**DOI:** 10.1038/bjc.1998.279

**Published:** 1998-05

**Authors:** R. P. Symonds

**Affiliations:** Beatson Oncology Centre, Western Infirmary, Glasgow, UK.

## Abstract

Mucositis may be a painful, debilitating, dose-limiting side-effect of both chemotherapy and radiotherapy for which there is no widely accepted prophylaxis or effective treatment. The basis of management is pain relief, prevention of dehydration and adequate nutrition. When tested vigorously, most antiseptic mouthwashes and anti-ulcer agents are ineffective. Simple mechanical cleansing by saline is the most effective traditional measure. A variety of new agents are effective. Granulocyte macrophage colony-stimulating factor (GM-CSF) and granulocyte colony-stimulating factor (G-CSF) act outwith the haemopoeitic system and can reduce mucositis, but the best schedule, dosage and method of administration is not known or which is the best growth factor to prevent this side-effect. A placebo-controlled randomized trial of antibiotic pastilles has shown a significant reduction in mucositis and weight loss during radiotherapy for head and neck cancer. Another method to reduce radiation effects in normal tissue is to stimulate cells to divide before radiotherapy by silver nitrate or interleukin 1. These methods may be particularly effective when given along with hyperfractionated radiation treatment such as CHART.


British Journal of Cancer (1998) 77(10), 1689–1695
© 1998 Cancer Research Campaign

### Treatment-induced mucositis: an old problem with new remedies

**RP Symonds**

Beatson Oncology Centre, Western Infirmary, Glasgow G11 6NT, UK

**Summary** Mucositis may be a painful, debilitating, dose-limiting side-effect of both chemotherapy and radiotherapy for which there is no widely accepted prophylaxis or effective treatment. The basis of management is pain relief, prevention of dehydration and adequate nutrition. When tested vigorously, most antiseptic mouthwashes and anti-ulcer agents are ineffective. Simple mechanical cleansing by saline is the most effective traditional measure. A variety of new agents are effective. Granulocyte macrophage colony-stimulating factor (GM-CSF) and granulocyte colony-stimulating factor (G-CSF) act outwith the haemopoeitic system and can reduce mucositis, but the best schedule, dosage and method of administration is not known or which is the best growth factor to prevent this side-effect. A placebo-controlled randomized trial of antibiotic pastilles has shown a significant reduction in mucositis and weight loss during radiotherapy for head and neck cancer. Another method to reduce radiation effects in normal tissue is to stimulate cells to divide before radiotherapy by silver nitrate or interleukin 1. These methods may be particularly effective when given along with hyperfractionated radiation treatment such as CHART.

**Keywords:** mucositis; prevention; radiotherapy; chemotherapy

Mucositis is a painful, debilitating, dose-limiting side-effect of both chemotherapy and therapeutic irradiation of the head and neck. The pathogenesis is straightforward: after chemotherapy or during radiotherapy, cells in the basal layers of the mucous membranes of the upper airways or upper digestive tact are unable to replace adequately cells lost through inactivation or exfoliation. Initially, there may be a transient white discoloration followed by deepening erythema and later a white pseudomembrane, which may be patchy or confluent. The most severe manifestation is ulceration of the mucosa (Holmes, 1991). The resultant mucosal damage may then be exacerbated by colonization of the affected area by abnormal bacterial flora. Mucositis may be aggravated by concomitant neutropenia and may be an important source of systemic infection (Berkowitz et al, 1983).

Some degree of mucositis is inevitable when radical doses of radiation are used to treat head and neck cancer. As a consequence, eating can become difficult, with an average weight loss of 5 kg during treatment (Symonds et al, 1996). Mucositis tends to occur in the third week of treatment. When patients are treated with so-called conventional-sized fractions of 2 Gy, mucositis can often peak in the fifth to sixth week of treatment and, in spite of continuing radiotherapy, mucositis can become less severe owing to accelerated repopulation of normal tissues. Mucositis may lead to premature end of a planned course of radiotherapy or a rest from treatment. Both actions can lead to tumour persistence (Amdur et al, 1989). The problem is particularly associated with accelerated hyperfractionated regimens. Mucosal reactions tend to peak earlier and are more severe but are of shorter duration than those after conventional treatment (Saunders et al, 1996). As these regimens are associated with improved local control, particularly in advanced disease (Horiot et al, 1997), any effective measure that can reduce this side-effect will be very useful to patients treated by twice or three times daily radiation regimens.

The problem is not restricted to radiation treatment. A whole variety of chemotherapeutic agents can have effects upon the oropharyngeal mucosa, including 5-fluorouracil with or without folinic acid, interferon, methotrexate, bleomycin, doxorubicin and epirubicin, cisplatin, vinblastine and the taxanes. Mucositis may be the dose-limiting toxicity with a variety of regimens. As many as 80% of patients receiving 5-fluorouracil and folinic acid may develop mucositis and, in up to 26%, this may be severe (Poon et al, 1989). Ulceration of the oropharynx with consequent dysphagia reaches its peak 10 or so days after chemotherapy. Concomitant neutropenia may encourage secondary colonization of the mouth with a wide variety of Gram-negative and anaerobic bacteria along with fungi, particularly *Candida albicans*. The problem is particularly marked during bone marrow transplantation. Stomatitis, secondary to myelosuppressive doses of chemotherapy, can not only be very painful and debilitating but may be the source of life-threatening sepsis. The origin of septicaemias is the mouth in 25–50% of immune-suppressed patients (Epstein et al, 1992). Almost all cases of systemic candidiasis originate from the oral cavity.

There is not a standard, widely accepted treatment to prevent or ameliorate chemotherapy- or radiation-induced mucositis. Traditionally, a range of remedies have been available to treat the acute side-effects of cancer treatment within the mouth and the pharynx. Only recently have some of these treatments been assessed rigorously and found to be wanting. However, there is a new range of novel therapies that alone or in combination can markedly reduce dose-limiting side-effects and improve the quality of life.

*Received 26 June 1997*
*Revised 10 September 1997*
*Accepted 23 October 1997*

*Correspondence to:* RP Symonds

### TRADITIONAL REMEDIES

Many traditional treatments are ineffective. However, the baby should not be thrown out with the bath water. The basic principles of mouth care stand the test of time. They are to relieve pain, prevent dehydration and provide adequate nutrition and deal with any focus of infection, such as obvious candidiasis. Good dental hygiene is paramount and the toothbrush is invaluable in reducing oral infection. Caries should be treated before anti-cancer treatment. A variety of mouthwashes are strongly recommended, often without any evidence to support this recommendation. Traditional remedies, such as glycerine and thymol and glycerine and lemon, are not effective (Hatton-Smith, 1994). Sodium bicarbonate mouthwashes can be harmful if too concentrated by altering oral pH (DeWalt and Haines, 1969), and hydrogen peroxide has been shown to be no more effective than a saline mouthwash (Feber, 1996). The antiseptic, chlorhexidine, and benzydamine, a non-steroidal inflammatory agent with anaesthetic and antimicrobial properties, are popular in the treatment of mucositis. However, neither have been shown to be effective. Patients receiving radiation therapy were randomized in a double-blind fashion to receive either chlorhexidine or a placebo mouthwash. The trend was for more mucositis and more toxicity in the chlorhexidine arm of the study. In this arm, more patients complained of mouthwash-induced discomfort, taste alteration and teeth staining. Owing to the degree of toxicity, it was felt that chlorhexidine was actually detrimental (Foote et al, 1994).

Chlorhexidine and benzydamine have been compared in a randomized trial, and there was no difference in either pain relief or mucositis. Altogether, 92% of the patients using benzydamine complained of pain in the mouth when using the mouthwash, and 50% refused to continue to use benzydamine (Samaranayake et al, 1988). A controlled trial of this agent compared with a placebo (10% alcohol) claimed that radiation mucositis was reduced. However, the benzydamine had to be diluted, and the local anaesthetic lidocaine was given in addition. The patients also received a variety of systemic analgesics that were not allowed for in the analysis (Epstein et al, 1989).

A three-arm trial in leukaemic patients of chlorhexidene vs chlorhexidine and nystatin showed plain saline to be as effective as the other two agents (Epstein et al, 1992). The nystatin may have been rendered ineffective by the chlorhexidine, as the combination has been shown to form a low-solubility salt. The minimum inhibitory concentration for candida is higher for the combination than for either drug alone (Barkvoll and Attramadal, 1989).

The message from all of these studies is that frequent mechanical cleansing of the mouth by a simple saline solution is the most effective measure, and more sophisticated mouthwashes may be positively harmful.

### ANTI-ULCER AGENTS

In severe mucositis, the mucous membrane can ulcerate; therefore, a logical approach was to test the use of agents that promoted the healing of ulcers and coated the mucous membrane to prevent further damage. Such an agent is sucralfate. This drug was first used to treat duodenal ulcers. It forms a potential barrier on damaged and normal mucosa and is largely unabsorbed. A phase 2 study of this disulphated disaccharide in combination with fluconazol suggested that mucositis with associated pain could be reduced markedly (Allison et al, 1995). However, randomized controlled trials (Barker et al, 1991; Makkonen et al, 1994; Epstein and Wong, 1994) have not shown that this agent prevents mucositis. It may, nevertheless, reduce oral discomfort to a limited extent.

Other anti-ulcer agents have been tried. Pentoxifylline has been found to be useful in the treatment of chronic trophic leg ulcers. However, a double-blind randomized placebo-controlled crossover trial did not show any effect in preventing mucositis in patients treated with cisplatin and 5-fluorouracil (Verdi et al, 1995).

Another agent, azelastine, has been shown to produce clinical improvement in aphthous ulcers and Behçet's disease. Those treated with radiotherapy, peplomycin and fluorouracil were treated with either azelastine, vitamin C, vitamin E and glutathione or the same regimen minus the azelastine. There is a suggestion that the addition of azelastine reduced mucositis (Osaki et al, 1994).

Feeding experimental animals additional glutamine seems to reduce the effects of cytotoxic agents, particularly 5-fluorouracil and methotrexate (O'Dwyer et al, 1987; Fox et al, 1988). Bowel ulceration caused by these anti-metabolites and corresponding bacteraemia was reduced (Fox et al, 1988). However, when additional glutamine supplements were given to patients suffering from gastrointestinal cancer, treated by 5-fluorouracil and folinic acid in a placebo-controlled double-blind trial, there was no difference in the degree of observed oral mucositis (Jebb et al, 1994).

Prostaglandins are cytoprotective compounds especially in the gastrointestinal tract. Topical applications have been shown to lead to healing of chronic leg ulcers (Eriksson et al, 1986). Intra-orally applied prostaglandin $E_2$ is not significantly absorbed (Matejka et al, 1990) but may reduce mucositis. Two very small, non-randomized studies claim a reduction in mucositis after both radiotherapy (Sinzinger et al, 1989) and chemotherapy (Kuhrer et al, 1986).

### Granulocyte and granulocyte macrophage colony-stimulating factors

Granulocyte colony-stimulating factor (G-CSF) and granulocyte macrophage colony-stimulating factor (GM-CSF) belong to a family of glycoprotein growth factors, which promote the proliferation and differentiation of neutrophil and monocyte/macrophage precursors and enhance the effector functions of mature neutrophils in vitro and in vivo. They are produced by stromal cells within the haemopoietic microenvironment (fibroblasts and endothelium) and by immunocompetent cells (T cells and macrophages). The action of these glycoproteins was thought to be restricted initially to haemopoietic cells only, but laboratory studies have shown that both G-CSF and GM-CSF influence the migration and proliferation of human endothelial cells, suggesting that these cytokines may act as regulatory signals outside the haemopoietic system (Bussolino et al, 1989). Clinical experience has also shown that these regulating proteins could stimulate the cells of the mucous membranes of the oropharynx. Twenty-seven patients with transitional cell carcinoma of the urogenital tract were treated with methotrexate, doxorubicin, vinblastine and cisplatin and were given G-CSF to reduce chemotherapy-induced neutropenia. Not only was there a reduction in the degree of neutropenia, but chemotherapy-induced mucositis was significantly reduced. Some 44% of patients who did not receive G-CSF developed mucositis compared with 11% of patients who had G-CSF during the first or second cycles of chemotherapy ($P = 0.041$) (Gabrilove et al, 1988). Mucositis secondary to high-dose cytarabine and mitoxantrone used to treat refractory non-Hodgkin's lymphoma was reduced when patients received GM-CSF. As well as the expected reduction in severe neutropenia, the incidence of mucositis was reduced from 60% in those patients not receiving GM-CSF to 17% with GM-CSF (Ho et al, 1990).

Mucositis is a particularly troublesome problem in patients receiving bone marrow transplantation. The incidence of

*© Cancer Research Campaign 1998*

mucositis can be as high as 100% in those receiving myeloablative chemotherapy and can be severe (grades 3 and 4) in 82% (Wardley and Scarffe, 1996). The severity of mucositis was reduced by GM-CSF in patients receiving thiotepa, total body irradiation and etoposide before bone marrow transplantation (Spadinger et al, 1994). A similar reduction in the severity of mucositis was seen after G-CSF in patients treated with cyclophosphamide, carmustine and etoposide before autologous bone marrow transplantation for Hodgkin's disease (Taylor et al, 1989). GM-CSF markedly reduced the incidence of stomatitis secondary to radiotherapy in patients receiving double half-body irradiation for refractory myelomatosis (Troussard et al, 1995). Patients receiving GM-CSF had only grade 1 stomatitis in comparison with previously treated patients, all of whom developed grade 3 or 4 stomatitis without the colony-stimulating factor. These phase 2 studies led to a series of randomized controlled trials. A total of 109 patients suffering from Hodgkin's disease, non-Hodgkin's lymphoma, a variety of leukaemias and myeloma were treated with myeloablative chemotherapy followed by an HLA-matched identical sibling marrow transplant. Fifty-three patients received GM-CSF, the other 56 received a placebo. There was a significant reduction in both grade 3 and 4 mucositis and infection in the GM-CSF-treated arm (Nemunaitis et al, 1995). A Japanese study (Katano et al, 1995) of only 14 patients receiving intra-arterial doxorubicin with or without G-CSF suggested that oral mucositis secondary to doxorubicin can be reduced or prevented by this cytokine.

A study of patients receiving chemotherapy for advanced head and neck cancer was particularly interesting and gave new information about the actions of GM-CSF. The response rates of regimens containing cisplatin, 5-fluorouracil and folinic acid for advanced head and neck cancer are high, but mucositis tends to be the dose-limiting toxicity. Twenty patients with stage 4 squamous carcinoma received cisplatin 20 mg $m^{-2}$ daily, 5-fluorouracil 800 mg $m^{-2}$ and folinic acid 90 mg $m^{-2}$ by continuous infusion over 96 h every 3 weeks. They were randomized to receive subcutaneous GM-CSF on days 5–14 after either the first or the second cycle of chemotherapy. Each patient therefore acted as their own control in this crossover study. After the first cycle of chemotherapy, the patients treated with the GM-CSF had a markedly reduced incidence of oral mucositis compared with those not receiving the growth factor. The incidence of severe mucositis was reduced when GM-CSF was given during the second cycle of chemotherapy. Interestingly, those patients who received GM-CSF with the first cycle of chemotherapy only appeared to have continued benefit from the colony-stimulating factor with reduction of mucositis in the second cycle of chemotherapy. This study goes some way to showing the mechanism of action of GM-CSF upon the oral mucosa. Patients with prolonged periods of neutropenia after chemotherapy are more likely to develop mucositis. The critical neutrophil count appears to be in the order of $1 \times 10^9\ l^{-1}$. The particular regimen of chemotherapy used in this study was fairly non-myelotoxic, and no patients had neutrophil counts falling below the critical level of $1 \times 10^9\ l^{-1}$. The incidence and severity of neutropenia was the same in patients with or without GM-CSF. The reduction of oral mucositis by GM-CSF in this study did not appear to be related to the granulocyte-stimulatory action of the growth factor. The inference is that the GM-CSF had a direct effect upon the oral mucosa (Chi et al, 1995).

There have been a number of negative studies in which the degree of mucositis has not been reduced by haemopoietic growth factor support. The degree of severe neutropenia was reduced when patients received G-CSF along with VAPEC-B chemotherapy (Pettengell et al, 1992) for non-Hodgkin's lymphoma. A greater dose intensity of chemotherapy could be given to these patients, but severe mucositis was the dose-limiting toxicity. Again, neutropenia was reduced when patients with advanced breast cancer received G-CSF along with 2-weekly high-dose doxorubicin and cyclophosphamide (Ferguson et al, 1993), but almost half the courses of chemotherapy were complicated by moderate to severe oral mucositis. The degree of mucositis was similar to historical controls in ten patients receiving HLA identical sibling marrow transplants for lymphoid malignancy, pretreated with high-dose busulphan and cyclophosphamide (Atkinson et al, 1991). Subcutaneous GM-CSF was given from day 7 to day 13 after transplantation. Interestingly, the incidence of graft vs host disease was worse in those patients receiving GM-CSF.

In all of these studies, the haemopoietic stimulating factor has been given either subcutaneously or intravenously. Currently, trials are underway with GM-CSF mouthwashes in patients receiving myeloablative chemotherapy (Wardley and Scarffe, 1996) and in the treatment of radiation mucositis both at the Radiumhemmet and in Greece (Throuvalas et al, 1995).

### ANTIBIOTICS

The oropharyngeal bacterial flora consists mainly of anaerobic bacteria, a lesser number of viridans streptococci and Neisseria species. Irradiation and local tumour surgery both interfere with the mucosal defences important for the maintenance of a microbiological balance. After surgery, there may be impaired motility of normal structures, and grafts may still be healing with areas of necrotic tissue, which may be colonized by abnormal bacterial flora. Necrotic tumour is a very good growth medium for many bacteria. Radiotherapy can encourage bacterial overgrowth in two ways. The first is by damaging and killing rapidly dividing cells in the oropharynx, leading to ulceration and colonization by abnormal bacteria. Saliva production may be reduced, as one or more parotid glands are often within the radiation field. Saliva washes away intraoral debris, food particles and bacteria and contains immunoglobin A, all factors important in the maintenance of normal mucosal bacterial flora. It has been suggested that micro-organisms, particularly aerobic Gram-negative bacteria, play a role in irradiation mucositis (van Saene and Martin, 1990). Although such bacteria are present in only very small numbers in healthy subjects, a Gram-negative bacillary carriage rate of 57% was found in patients before irradiation (Spijkevet et al, 1989). The frequency of bacillary carriage in this group of patients led the same group to try a novel approach to the problem (Spijkevet et al, 1991). Lozenges containing polymyxin E, tobramycin and amphotericin B were given to a group of 15 patients treated by radiotherapy for head and neck cancer. Mucositis was confined to erythema only in these patients. A much larger placebo-controlled double-blind trial of similar antibiotic pastilles (Symonds et al, 1996) showed a clinically beneficial effect, although less striking than the smaller study of Spijkevet. A total of 275 patients suffering from T1–T4 head and neck cancer were entered into the study. Of these, 136 patients were allocated to suck four times a day a pastille containing amphotericin B, polymyxin and tobramycin. The remaining 139 patients received an identical placebo. Bacteriological monitoring was carried out before and twice weekly during treatment. Both arms of the study were well balanced for T and N stage, age, sex and radiation dose (60 Gy).

© Cancer Research Campaign 1998

There was a reduction in mucositis distribution ($P$ = 0.002), mucositis area ($P$ = 0.028), dysphagia ($P$ = 0.006) and, especially, weight loss ($P$ = 0.009), which is a highly objective end point. There was a clear tendency for patients with positive cultures for aerobic, Gram-negative bacteria ($P$ = 0.003) and yeasts ($P$ = 0.026) during treatment to have more severe mucositis. The active pastilles reduced the percentage of patients with yeast cultures ($P$ = 0.003) but had less effect on aerobic Gram-negative bacteria.

A smaller study from the Mayo Clinic of similar antibiotic lozenges showed no difference in mucositis scores measured by a nurse or radiation oncologist, but the mean patient mucositis score and the duration of patients reporting grade 3 and 4 mucositis were both lower in the antibiotic lozenge arm of the study ($P$ = 0.02 and 0.007 respectively) (Okuno et al, 1997).

In patients receiving allogenic bone marrow transplantation, up to 64% of septicaemias originate from oral infection (Donnelly et al, 1992). As in the case of patients with oral cancer, immunosuppressed patients have an increased frequency of carriage of aerobic Gram-negative bacteria and fungi (Woo et al, 1993). The use of prophylactic antibiotics (Meissenberg et al, 1996) certainly reduces the incidence of fever and bacteraemia in this group of patients, but the impact on mucositis is less well documented. Intuitively, one would expect systemic antibiotics to have some effect on mucositis.

### NOVEL AGENTS

Capsaicin, the active ingredient in chilli peppers, has been shown to provide temporary pain relief in 11 patients with oral mucositis secondary to either chemotherapy or radiotherapy (Berger et al, 1995). The vehicle for the capsaicin was candy, and the authors list their own 'taffy' recipe. These patients took two to six candies a day for continuous pain relief. One problem with capsaicin is an initial burning sensation in the mouth. This was dealt with by giving concomitant benzocaine.

Ice in the mouth can cause local vasoconstriction and may reduce the uptake of 5-fluorouracil into mucosal cells. As the plasma half-life of this drug is short (5–20 min), patients were asked to suck ice chips for 5 min before and 30 min after 425 mg m$^{-2}$ 5-Fu along with folinic acid 20 mg m$^{-2}$ i.v. given for 5 days. A total of 95 patients were randomized to suck ice or to serve as a control group during the first cycle of chemotherapy and subsequently crossed over in the next cycle. Mucositis was reduced significantly ($P$ = 0.002). Apart from numbness of the mouth and 'ice cream' headaches, the ice was well tolerated (Mahood et al, 1991).

A follow-on study (Rocke et al, 1993) showed that using ice for 60 min offered no extra protection. Ice packs have also been shown to reduce 5-Fu-induced ocular irritation (Loprinzi et al, 1994).

Melphalan-induced stomatitis may also be prevented by cryotherapy. In a series of 18 patients, only one developed grade 3 mucositis when ice pops were sucked 5 min before and stopped 5 min after a melphalan infusion (140–180 mg m$^{-2}$ over 30 min) before marrow transplantation (Meloni et al, 1966).

At the start of a course of radiotherapy treatment, a considerable number of cells within the basal layer of the buccal mucosa are not undergoing division. Initially, there may be a decrease in mitotic activity, leading to the retention of superficial cells, which appear white owing to the greater degree of keratinization. Radiation-induced leukoplakia is one of the earliest changes affecting the oral cavity, especially the mucosa and dorsum of the tongue (Fine, 1974). As more cells are shed from the mucosa, the rate of division of the basal layer increases rapidly to replace cell loss (Holmes, 1991). During the first week of radiotherapy, there may be a discrepancy between cell proliferation and cell killing. This is especially true in patients receiving hyperfractionated accelerated radiotherapy. It has been suggested that cell production could be stimulated before radiotherapy by the use of silver nitrate. Sixteen patients suffering from advanced oral cancer were treated by accelerated hyperfractionation. This was a split-course schedule of 1.6 Gy twice daily five times a week initially, to a total dose of 32 Gy. After a gap of 9–12 days (depending on the severity and healing of acute mucositis), patients were treated again twice daily to a total dose of 66–74 Gy. Five days before radiotherapy and for the first two days of treatment, the left side of the patient's mouth was brushed with 2% silver nitrate three times a day after meals, and the right side was left untreated to act as a symmetrical individual control. The degree of mucositis appeared to be greater on the untreated side of the mouth. Most patients had confluent mucositis by the end of the first part of the radiotherapy in this area. By comparison, the reaction was no worse than severe erythema in the silver nitrate-treated area (Maciejewski et al, 1992).

One of the methods by which GM-CSF may stimulate oral mucosal cells is by enhancing interleukin 1 (IL-1) transcription and translation (Dinarello, 1991). Interleukin 1 has been shown to convey substantial protection to normal cells against the lethal and sublethal effects of whole-body irradiation, extensive chemotherapy and localized irradiation in experimental animals (Zaghloul et al, 1994). It has been suggested that the cytoprotective effects may result from increasing proliferation rates in mucosal cells. It has been shown particularly in experiments on irradiated mice in which pretreatment with IL-1 increased the thymidine labelling index particularly in the lip, tongue and crypt cells of the duodenum in irradiated mice. It is possible that local applications of IL-1 may increase proliferation rates before radiotherapy. It has been shown that endogenous IL-1 has the ability to trigger endogenous production of this substance in the epithelial cells cultured in vitro, which in turn can lead to higher levels of circulating IL-1 (Warner et al, 1987).

### Pilocarpine

A late oral complication of high-dose radiotherapy is xerostomia. Following irradiation of usually both parotid glands, there is permanent impairment of salivary production. As a consequence, there is marked susceptibility to dental caries and oral infections. Speaking, chewing and swallowing are more difficult. There is no effective means of treating a dry mouth secondary to salivary gland dysfunction (Fox et al, 1991), and many patients carry a small bottle of water.

Pilocarpine is a parasympathetic stimulant of exocrine secretion. Two placebo-controlled double-blind trials have shown the value of this alkaloid (Johnson et al, 1993; LeVeque et al, 1993). Both multicentre studies were similar in design and contained 207 and 162 patients respectively. At least one parotid gland had been irradiated to doses above 40 Gy, but patients had to have some evidence of residual salivary function. Salivary production was measured and symptoms were assessed by a visual analogue scale. In both studies, pilocarpine improved symptoms compared with a placebo, but in only one (LeVeque et al, 1993) was salivary production increased to a statistically significant degree, although the volume increase was small. It is possible that qualitative differences in saliva production rather than quantitative differences

© Cancer Research Campaign 1998

helped to improve symptoms, particularly production of more mucin, which is a more long-lasting lubricant and mucosal wetting agent. A dose of 5 mg three times daily was effective in most patients, and adverse effects were usually limited to increased sweating.

### CONCLUSION

Many of the traditional remedies for mucositis can be discarded as they have been shown to be ineffective or even harmful. The most effective measure is frequent oral rinsing with a bland mouthwash, such as saline, to reduce intraoral bacteria. Good dental hygiene is essential. The importance of the reduction of plaque and the control of periodontal disease is often forgotten. Before radiotherapy of the head and neck or, for that matter, chemotherapy likely to produce mucositis, such as before bone marrow transplantation, treatment of pre-existing caries and the removal of any pre-existing calculus is vital, as pre-existing dental infection can be a potent source of systemic infection. Even in health, bacteraemia after oral and dental procedures is not at all uncommon (McElroy, 1984). Patients should be encouraged to use a soft toothbrush and to use dental floss regularly.

Topical non-absorbable antibiotics do appear to reduce the unpleasant sequelae of radiation treatment for head and neck cancer, although neither the optimal type of antibiotics nor the method of administration has been worked out. Use of antibiotic pastilles was strikingly less effective in the reduction of aerobic Gram-negative bacteria than when the same antibacterials were applied as a gel or a paste to the buccal mucosa in intensive care patients, when aerobic Gram-negative bacteria were reduced drastically within 3–4 days (Stoutenbeek et al, 1984; Ledingham et al, 1988). There seems to be a need for new formulations to allow the protracted delivery of antimicrobials to the oropharynx in patients receiving therapeutic irradiation.

Interleukin 1 and silver nitrate appear to improve the tolerance of experimental animals and patients with head and neck cancer, respectively, to radiotherapy by increasing the number of cycling cells before this treatment. Both GM-CSF and G-CSF probably act in the same way in the case of radiation treatment. The precise timing of these colony-stimulating factors in the case of chemotherapy is much more problematical and may well depend on the agents used and the schedule in which they are given. Mucositis is usually the dose-limiting toxicity of patients receiving 5-fluorouracil and folinic acid. They were given concomitant G-CSF (5 $\mu g\ kg^{-1}\ day^{-1}$ subcutaneously for 14 days) along with 5-fluorouracil (425 $mg/m^{-2}$) and folinic acid (20 $mg\ m^{-2}$) for 5 days repeated every 28 days. Four out of five assessable patients treated with this regimen developed grade 4 myelosuppression. Other patients were treated with the same regimen but the G-CSF administration began 24 h after the last dose of 5-fluorouracil. None of these patients developed neutropenia. It was felt that the concurrent administration of the haematological growth factor was responsible for the increased toxicity of chemotherapy. Interestingly, although the aim of the study initially was to see if mucositis could be reduced by this approach, the authors do not comment on the degree of mucositis in treated patients in their publication (Meropol et al, 1992). The scheduling of the cytokine could be responsible for either the success or the failure in preventing mucositis. Chi and colleagues (1995) illustrated a marked reduction in chemotherapy-induced mucositis in a crossover study. The chemotherapy was given over 5 days and repeated every 3 weeks with GM-CSF beginning at the end of chemotherapy. The two studies that stand out as showing a negative effect for mucositis reduction by G-CSF have similar characteristics. In both, intensive chemotherapy was given frequently along with G-CSF. The first is a study of VAPEC-B chemotherapy in which doxorubicin in combination with cyclophosphamide or etoposide was given every 2 weeks. In between, vincristine and bleomycin were administered. Bleomycin in particular can be quite toxic to the oral mucosa (Pettengell et al, 1992). In the other study, doxorubicin (100 $mg\ m^{-2}$) or doxorubicin and cyclophosphamide were given at 2-weekly intervals (Ferguson et al, 1993). A 10-day course of G-CSF was started 24 h after each cycle of chemotherapy. The VAPEC-B patients received daily subcutaneous G-CSF for up to a week preceding and for up to 2 weeks after treatment with doxorubicin, cyclophosphamide and etoposide. The combination of the cytokine and the chemotherapy seemed to have a definite beneficial effect upon the neutrophil count but may not be optimal for preventing mucositis. Most studies of the cytokines have been directed towards their effect upon the haemopoietic system, and dosages and schedules are usually tailored accordingly. The optimal doses for preventing mucositis are not known; neither is the method of delivery (subcutaneous or intravenous) and the duration and timing of treatment. It is not known which is the superior cytokine, either G-CSF or GM-CSF.

The use of GM-CSF mouthwashes is an intriguing development. On the little evidence available, perhaps such mouthwashes should be administered before fractionated radiotherapy in order to promote cell division, so that cell replication in the mouth can keep up with cell killing by radiotherapy. The timing of the use of mouthwashes with chemotherapy is far less clear and would seem to depend on the type of chemotherapy and how frequently these agents are given. It is possible that stimulation of mucosal cells to divide renders them more vulnerable to chemotherapy.

The greatest scope for reduction of mucositis is during accelerated hyperfractionated radiotherapy, such as the MRC CHART regimen. In this study, there was a trend for improved local control of advanced tumours, particularly laryngeal cancer (Saunders et al, 1996). Acute skin reactions were less than with conventional regimens, as was late treatment-related morbidity.

If acute mucositis could be reduced, a higher radiation dose could be given, leading to better local control and survival.

### REFERENCES

Allison RR, Vongtama V, Vaughan J and Shin KH (1995) Symptomatic acute mucositis can be minimised or prophylaxed by the combination of sucralfate and fluconazole. *Cancer Invest* **13**: 16–22

Amdur RJ, Parsons JT, Mendenhall WM, Million RR and Cassisi NJ (1989). Split course versus continuous irradiation in the post operative setting for squamous carcinoma of head and neck. *Int J Radiat Oncol Biol Phys* **17**: 279–285

Atkinson K, Biggs JC, Downs K, Juttner C, Bradsrock K, Lowenthal RM, Dale B and Szer J (1991) GM-CSF after allogenic bone marrow transplantation: accelerated recovery of neutrophils, monocytes and lymphocytes. *Aust NZ J Med* **21**: 686–692

Barker G, Loftus L, Cuddy P and Barker B (1991) The effects of sucralfate suspension and diphenhydramine syrup plus kaolin-pectin on radiotherapy induced mucositis. *Oral Surg Oral Med Oral Pathol* **71**: 288–293

Barkvoll P and Attramadal A (1989). Effect of nystatin and chlorhexidine digluconate on *Candida albicans*. *Oral Surg Oral Med Oral Path* **67**: 279–281

Berger A, Henderson M, Nadoolman W, Duffy V, Cooper D, Saberski L and Bartoshuk L (1995) Oral caspsaicin provides temporary relief for oral mucositis pain secondary to chemotherapy/radiation therapy. *J Pain Symptom Manage* **10**: 243–248

© Cancer Research Campaign 1998

Berkowitz RJ, Crock J and Strickland R (1983) Oral complications associated with bone marrow transplantation in a paediatric population. *Am J Pediat Haematol Oncol* **5**: 53–57

Bussolino F, Wang JM, Defilippi P, Turrini F, Sanavio F, Edgell CJS, Aglietta M, Arese P and Mantovani A (1989) Granulocyte and granulocyte-macrophage colony stimulating factors induce human endothelial cells to migrate and proliferate. *Nature* **337**: 471–473

Chi KH, Chen CH, Chan WK, Chow KC, Chen SY, Yen SH, Chao JY, Chang CY and Chen KY (1995) Effect of granulocyte-macrophage colony stimulating factor on oral mucositis in head and neck cancer patients after cisplatin, fluorouracil and leucovorin chemotherapy. *J Clin Oncol* **13**: 2620–2628

DeWalt E and Haines AK (1969). The effects of specified stressors on health of oral mucosa. *Nurs Res* **18**: 22–27

Dinarello CA (1991) Interleukin 1 and interleukin antagonism. *Blood* **77**: 1627–1652

Donnelly JP, Muus P and Schattenberg A (1992) A scheme for monitoring oral mucositis in allogenic BMT recipients. *Bone Marrow Transplant* **9**: 409–413

Epstein JB and Wong FLW (1994). The efficacy of sucralfate suspension in the prevention of oral mucositis due to radiation therapy. *Int J Radiat Oncol Biol Phys* **28**: 693–698

Epstein J, Stevenson-Moore P, Jackson S, Mohamed JH and Spinelli JJ (1989) Prevention of oral mucositis in radiation therapy: a controlled study with benzydamine hydrochloride rinse. *Int J Radiat Oncol Biol Phys* **16**: 1571–1575

Epstein JB, Vickars L, Spinelli J and Reece D (1992) Efficacy of chlorhexidine and nystatin rinses in prevention of oral complications in leukaemia and bone marrow transplantation. *Oral Surg Oral Med Oral Pathol* **73**: 682–689

Eriksson G, Johansson C, Aly C and Topical A (1986) Prostaglandin $E_2$ for chronic leg ulcers. *Lancet* **I**: 905

Feber T (1996) Management of mucositis in oral irradiation. *Clin Oncol* **8**: 106–111

Ferguson JE, Dodwell DJ, Seymour AM, Richards MA and Howell A (1993) High dose, dose intensive chemotherapy with doxorubicin and cyclophosphamide for the treatment of advanced breast cancer. *Br J Cancer* **67**: 825–829

Fine L (1995) Dental care of the irradiated patient. *J Hosp Dent Pract* **9**: 127–132

Foote RL, Loprinzi CL, Frank AR, O'Fallon JR, Gulavita S, Tewfik HH, Ryan MA, Earle JM and Novotny P (1994) Randomised trial of a chlorhexidine mouthwash for the alleviation of radiation induced mucositis *J Clin Oncol* **12**: 2630–2633

Fox AD, Kripke SA, De Paula JA, Berman JM, Settle RG and Rombeau JL (1988) Effect of a glutamine supplemented enteral diet on methotrexate induced enterocolitis. *J Parent Ent Nutr* **12**: 325–331

Fox PC, Atkinson JC, Macynski AA, Wolff A, Kung DS, Valdez IH, Jackson W, Delapenha RA, Shiroky J and Baum BJ (1991) Pilocarpine treatment of salivary gland hypofunction and dry mouth (xerostomia). *Arch Intern Med* **151**: 1149–1152

Gabrilove JL, Jakubowski A, Scher H, Sternberg C, Wong G, Grous J, Yagoda A, Fain K, Moore MAS, Clarkson B, Oettgen HF, Alton K, Welte K and Souza L (1988) Effect of granulocyte colony stimulating factor on neutropenia and associated morbidity due to chemotherapy for transitional cell carcinoma of the urothelium. *N Engl J Med* **318**: 1414–1422

Hatton-Smith CK (1994). A last bastion of ritualised practise? A review of nurses knowledge of oral healthcare. *Prof Nurse* **9**: 304–308

Ho AD, Del Valle F, Haas R, Engelhard M, Hiddemann W, Ruckle H, Schlimok G, Thiel E, Andreesen R, Fiedler W, Frisch J, Schulz G and Hustein W (1990) Sequential studies on the role of mitoxantrone, high dose cytarabine and recombinant human granulocyte-macrophage colony stimulating factor in the treatment of refractory non Hodgkin's lymphoma. *Semin Oncol* **17** (6, suppl. 10): 14–19

Holmes S (1991) The oral complications of specific anticancer therapy. *Int J Nurs Stud* **28**: 343–360

Horiot JC, Bontemp P, van den Bogaert W, Le Fur R, van de Weijngaert D and Bolla M (1997) Accelerated fractionation (AF) compared to conventional fractionation (CF) improves loco-regional control in the radiotherapy of advanced head and neck cancers: results of EORTC 22851 randomised trial. *Radiother Oncol* **44**: 111–121

Jebb SA, Osborne RJ, Maughan TS, Mohideen N, Mack P, Mort D, Shelley MD and Elia M (1994) 5 fluorouracil and folinic acid induced mucositis: no effect of oral glutamine supplementation. *Br J Cancer* **70**: 732–735

Johnson JT, Ferretti GA, Nethery WJ, Valdez IH, Fox PC, Ng D, Muscoplat CC and Gallagher SC (1993) Oral pilocarpine for post-irradiation xerostomia in patients with head and neck cancer. *N Engl J Med* **6**: 390–395

Katano M, Nakamura M, Matsuo T, Iyama A and Hisatsugu T (1995) Effect of granulocyte colony stimulating factor (G-CSF) on chemotherapy induced oral mucositis. *Surg Today Jpn J Surg* **25**: 207–210

Kuhrer I, Kuzmits R, Linkesch W and Ludwig H (1986) Topical PGE2 enhances healing of chemotherapy associated mucosal lesions. *Lancet* **1**: 622

Ledingham IMcA, Alcock SR, Eastaway AT, McDonald JC, McKay IC and Ramsay G (1988) Triple regimen of decontamination of the digestive tract, systemic cefotaximine and microbiological surveillance for prevention of acquired infection in intensive care. *Lancet* **1**: 785–790

LeVeque FG, Montgomery M, Potter D, Zimmer MB, Rieke JW, Steiger BW, Gallager SC and Muscoplat CC (1993) A multicenter, randomized, double-blind placebo-controlled, dose-titration study of oral pilocarpine for treatment of radiation induced xerostomia in head and neck patients *J Clin Oncol* **11**: 1124–1131

Loprinzi CL, Wender DB, Veeder MH, O'Fallon JR, Vaught NL, Dose AM, Ghosh C, Bartel J and Leitch JM (1994) Inhibition of 5-fluoruoracil-induced ocular irritation by ocular ice packs. *Cancer* **74**: 945–948

McElroy TH (1984). Infection in the patient receiving chemotherapy for cancer: oral candidiasis. *J Am Dent Assoc* **109**: 454–456

Maciejewski B, Zajusz A, Pitecki B, Skladowski Z, Dorr W, Kummermehr J and Trott KR (1992) Escalated hyperfractionation and stimulation of acute mucosal reaction in radiotherapy for cancer of oral cavity and oropharynx. *Semin Radiat Oncol* **2**: 54–57

Mahood DJ, Dose AM, Loprinzi CL, Veeder MH, Athmann LM, Therneau TM, Sorensen JM, Gainey DK, Mailliard JA, Gusa NL, Finck GK, Johnson C and Goldberg RM (1991) Inhibition of flourouracil-induced stomatitis by oral cryotherapy. *J Clin Oncol* **9**: 449–452

Makkonen TA, Bostron P, Vilja P and Joensuu H (1994) Sucralfate mouth washing in the prevention of radiation induced mucositis: a placebo-controlled double blind randomised study. *Int J Radiat Oncol Biol Phys* **30**: 177–182

Matejka M, Nell A, Kment A, Schein A, Leukauf M, Porteder H, Mailath G and Sinzinger H (1990) Local benefits of prostaglandin E2 in radiochemotherapy induced oral mucositis. *Br J Oral Maxillofac Surg* **28**: 89–91

Meisenberg B, Gollard R, Brehm T, McMillan R and Miller W (1996). Prophylactic antibiotics eliminate bacteraemia and allow safe outpatient management following high dose chemotherapy and autologous stem cell rescue. *Supportive Care Cancer* **4**: 364–369

Meloni G, Capria S, Proia A, Trisolini SM and Mandelli F (1996) Ice pops to prevent melphalan-induced stomatitis. *Lancet* **347**: 1691–1692

Meropol NJ, Miller LL, Korn EL, Braitman LE, McDermott ML and Schuchter LM (1992) Severe myelosuppression resulting from concurrent administration of granulocyte colony stimulating factor and cytotoxic chemotherapy. *J Natl Cancer Inst* **84**: 1201–1203

Nemunaitis J, Rosenfeld CS, Ash R, Freedman MH, Deeg HJ, Appelbaum F, Singer JW, Flomenberg N, Dalton W, Elfenbein GJ, Rifkin R, Rudin A, Agosti J, Hayes FA, Holcenberg J and Shadduck PK (1995) Phase 111 randomised double blind placebo controlled trial of rh GM-CSF following allogenic bone marrow transplant. *Bone Marrow Transplant* **15**: 949–954

O'Dwyer ST, Scott T, Smith RJ and Wilmore DW (1987) 5 Fluorouracil toxicity on small intestine but not white blood cells is decreased by glutamine. *Clin Res* **35**: 367A

Okuno SH, Foote RL, Loprinzi CL, Gulavita S, Sloan JA, Earle J, Novotny PJ, Burk M and Frank AR (1997) A randomized trial of a nonabsorbable antibiotic lozenge given to alleviate radiation-induced mucositis. *Cancer* **79**: 2193–2199

Osaki T, Ueta E, Yoneda K, Hirota J and Yamamoto T (1994) Prophylaxis of oral mucositis associated with chemoradiotherapy for oral carcinoma by azelastine hydrochloride (azelastine) with other antioxidants. *Head Neck* **16**: 331–339

Pettengell R, Gurney H, Radford JA, Deakin DP, James R, Wilkinson PM, Kane K, Bentley J and Crowther D (1992) Granulocyte colony stimulating factor to prevent dose limiting neutropenia in non Hodgkin's lymphoma: a randomised controlled trial. *Blood* **80**: 1430–1436

Poon MA, O'Connell MJ, Moertel CG, Wieand HS, Everson LK, Krook JE, Maillard JA, Laurie JA, Tscetter P and Weisenfeld W (1989) Biochemical modulation of fluorouracil: evidence of significant improvement of survival and quality of life in patients with advanced colo-rectal carcinoma. *J Clin Oncol* **7**: 1407–1418

Rocke LK, Loprinzi CL, Lee JK, Kunselman SJ, Iverson RK, Fuick G, Lifsey D, Glaw KC, Stevens BA and Hatfield AK (1993) A randomized clinical trial of two different durations of oral cryotherapy for prevention of 5-fluoruoracil-related stomatitis. *Cancer* **72**: 2234–2238

Samaranayake LP, Robertson AG, MacFarlane TW, Hunter IP, MacFarlane G, Soutar DS and Ferguson MM (1988) The effect of chlorhexidine and benzydamine on mucositis induced by therapeutic irradiation. *Clin Radiat* **39**: 291–294

Saunders MI, Dische S, Barrett A, Parmar MKB, Harvey A and Gibson D (1996) Randomised multicentre trials of CHART vs conventional radiotherapy in head and neck and non small cell lung cancer: an interim report. *Br J Cancer* **73**: 1455–1562

© Cancer Research Campaign 1998

Sinzinger H, Porteder H, Matejka M and Peskar B (1989) Prostaglandins in irradiation induced mucositis. *Lancet* **1**: 556

Spadinger BG, Hodges E, Ruby E, Stanley R and Coccia P (1994). Effect of granulocyte-macrophae colony stimulating factor on oral mucositis after haematopoietic stem cell transplantation. *J Clin Oncol* **12**: 1917–1922

Spijkervet FKL, van Saene HKF, Panders AK, Vermey A and Mehta DM (1989) Colonisation index of the oral cavity: a novel technique for monitoring a colonisation defence. *Microbiol Ecol Health Dis* **2**: 145–151

Spijkervet FKL, van Saene HKF, van Saene JJM, Panders AK, Vermey A and Mehta DM (1991) Mucositis prevented by selective elimination of oral flora in irradiated head and neck cancer patients. *J Oral Pathol Med* **19**: 486–489

Stoutenbeek CHP, van Saene HKF, Misanda DR, van der Waaiy DF and Zandstra DF (1984) The effect of selective decontamination of the digestive tract on colonisation and infection rate in multiple trauma patients. *Intensive Care Med* **10**: 185–192

Symonds RP, McIlroy P, Khorrami J, Paul J, Pyper E, Alcock SR, McCallum I, Speekenbrink ABJ, McMurray A, Lindemann E and Thomas M (1996) The reduction of radiation mucositis by selective decontamination antibiotic pastilles: a placebo controlled double blind trial. *Br J Cancer* **74**: 312–317

Taylor KM, Jagannath S, Spitzer G, Spinolo JA, Tucker SL, Fogel B, Cabanillas FF, Hagemeister FB and Souza LM (1989) Recombinant human granulocyte colony stimulating factor hastens granulocyte recovery after high dose chemotherapy and autologous bone marrow transplantation in Hodgkin's disease. *J Clin Oncol* **7**: 1791–1799

Throuvalas N, Antonadou D, Pulizzi M and Sarris G (1995) Evaluation of the efficacy and safety of GM-CSF in the prophylaxis of mucositis in patients with head and neck cancer treated by RT. In *Proceedings of European Conference of Clinical Oncology (ECCO)*, p. 593. Federation of European Cancer Societies: Paris (abstract 431)

Troussard X, Macro M, Vie B, Bastio A, Peny AM, Reman O, Tabah I and Leporrier M (1995) Human recombinant granulocyte macrophage colony stimulating factor (hr GM-CSF) improves double hemibody irradiation (DHBI) tolerance in patients with stage 111 multiple myeloma: a pilot study. *Br J Haematol* **89**: 191–195

van Saene HK and Martin MV (1990) Do micro-organisms play a role in irradiation mucositis? *Eur J Clin Microbiol Infect Dis* **9**: 861–863

Verdi CJ, Garewal HS, Koenig LM, Vaughn B and Buckhead T (1995) A double blind randomised placebo controlled, crossover trial of pentoxifylline for the prevention of chemotherapy induced oral mucositis. *Oral Surg Oral Med Oral Pathol Oral Radiol Endod* **80**: 36–42

Wardley AM and Scarffe (1996) Role of granulocyte macrophage colony stimulating factor in chemoradiotherapy induced oral mucositis. *J Clin Oncol* **14**: 1741–1742

Warner SJ, Auger KR and Libby P (1987) Interleukin 1 induces interleukin 1 gene expression in human vascular smooth muscle cells. *J Exp Med* **165**: 1316–1324

Woo SB, Sounis ST and Monopoli MM (1993) A longitudinal study in oral ulcerative mucositis in bone marrow transplant recipients. *Cancer* **72**: 1612–1617

Zaghloul MS, Dorie MJ and Kallman RF (1994) Interleukin 1 increases thymidine labelling index of normal tissues of mice but not the tumour. *Int J Radiat Oncol Biol Phys* **29**: 805–811

© Cancer Research Campaign 1998 


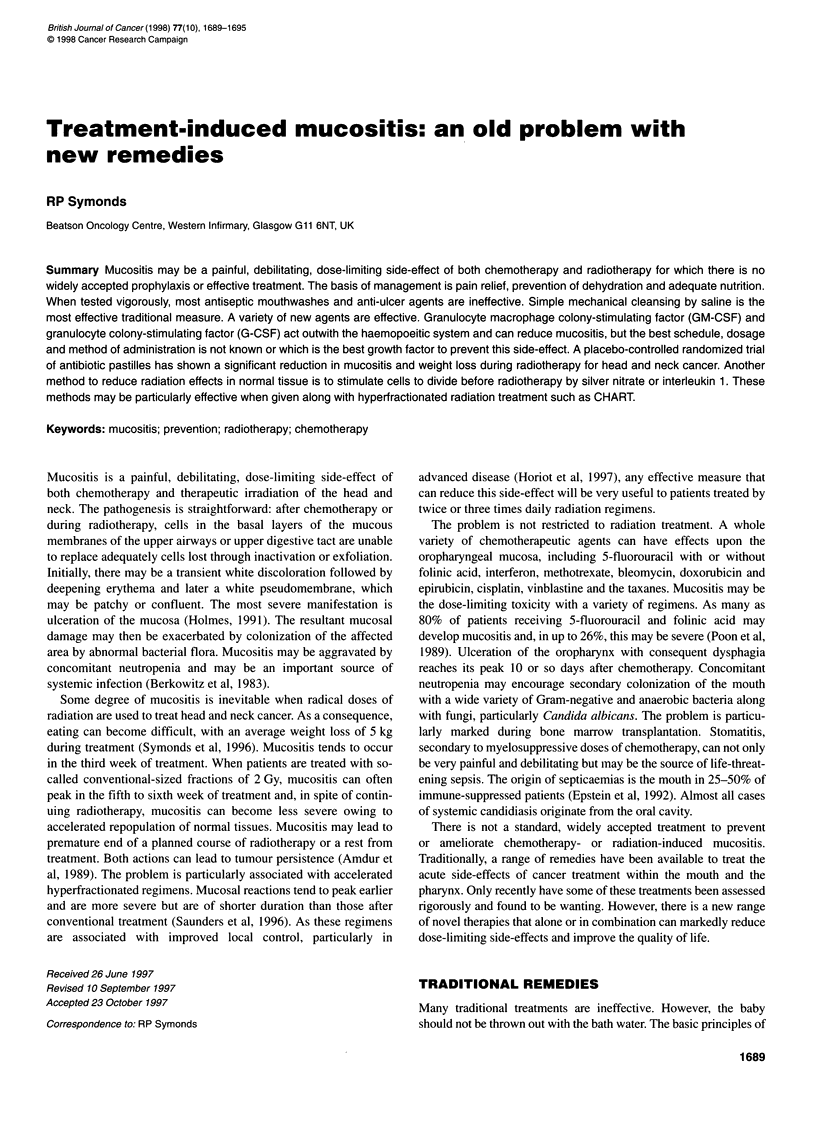

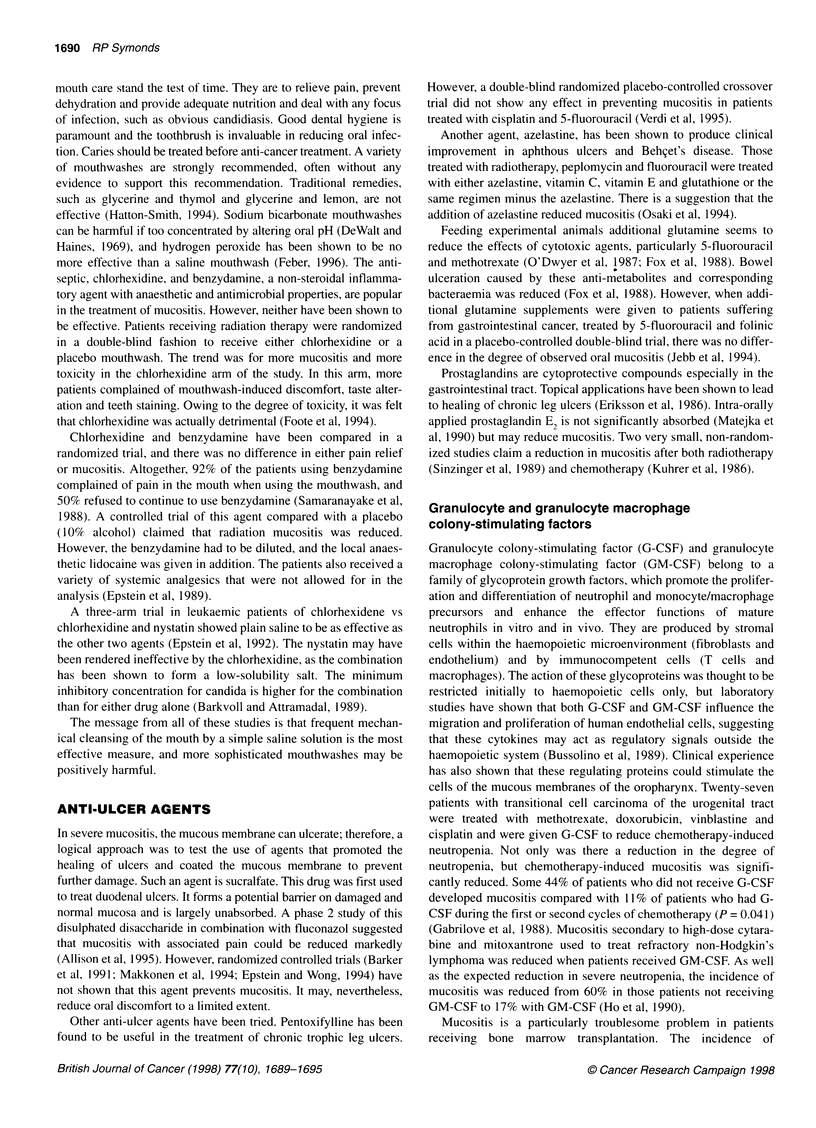

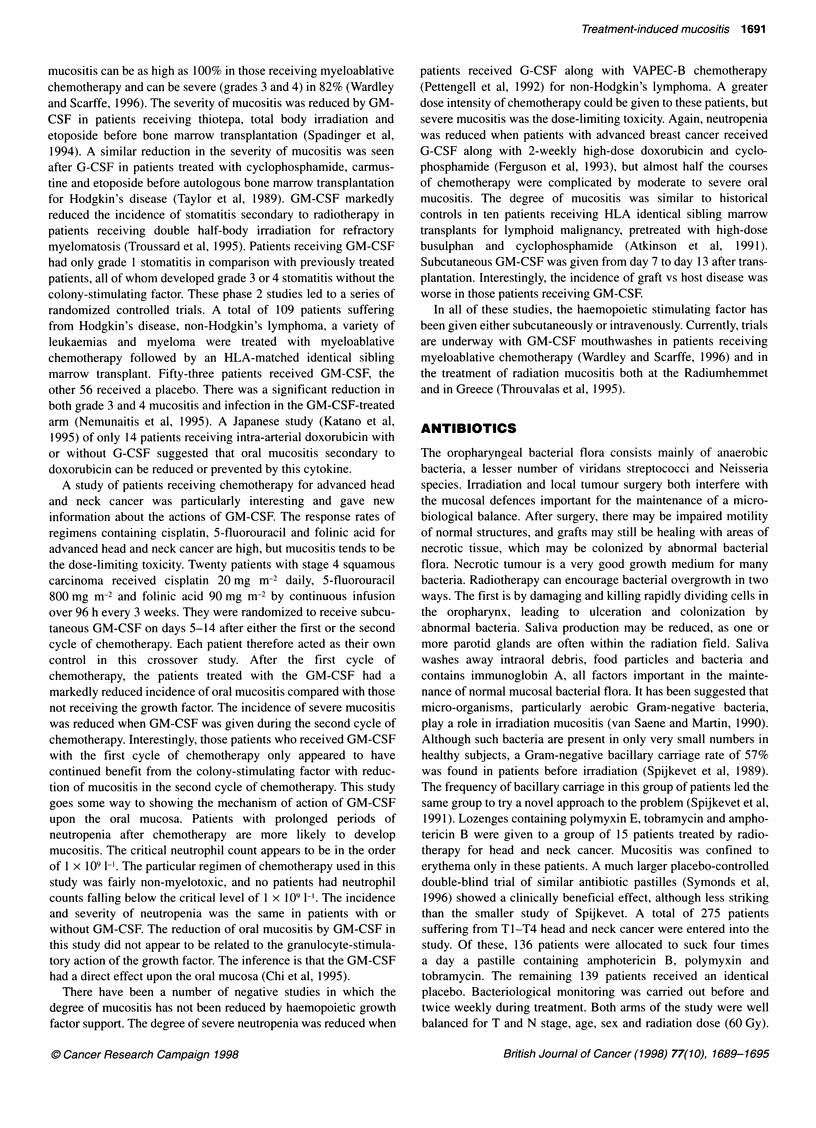

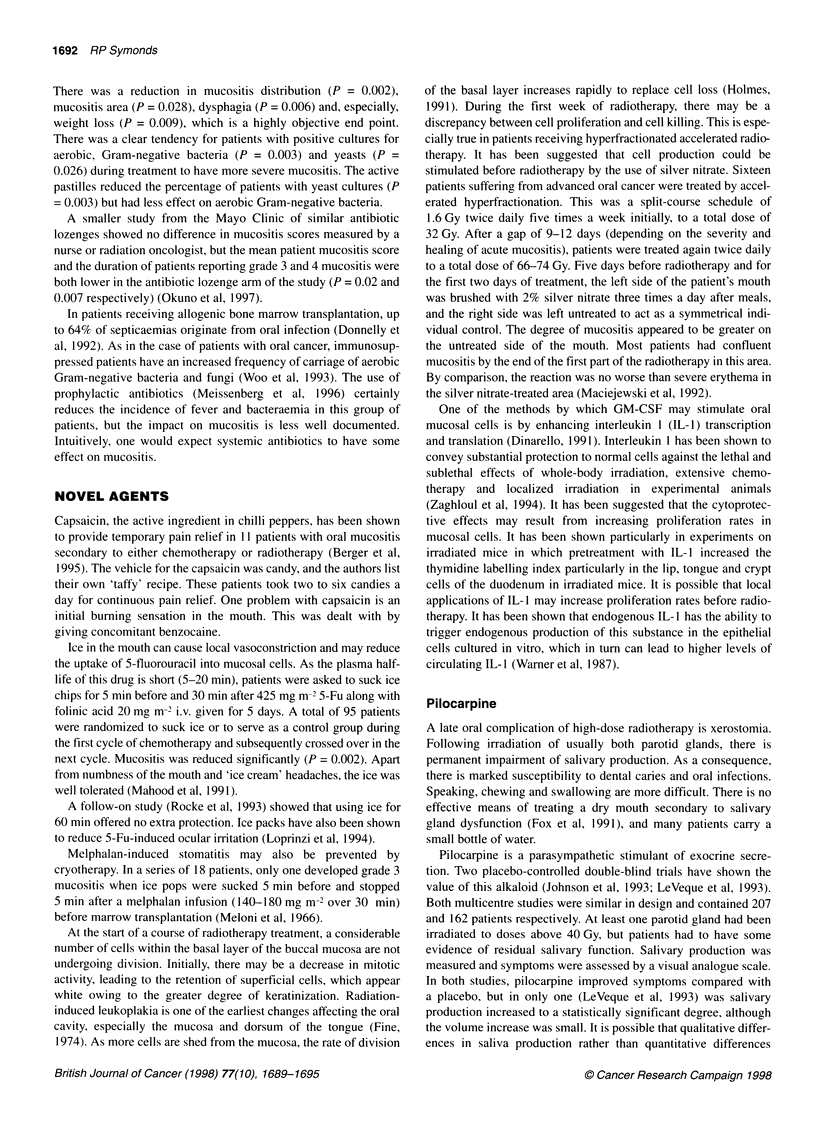

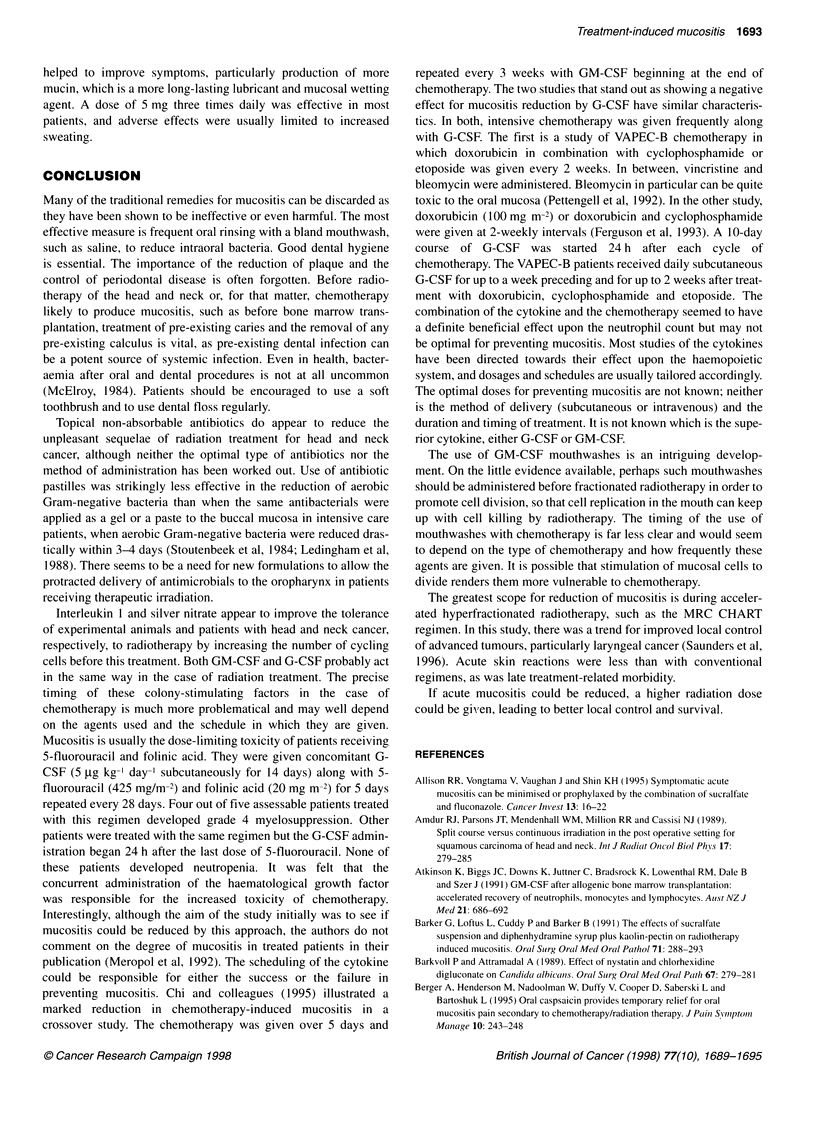

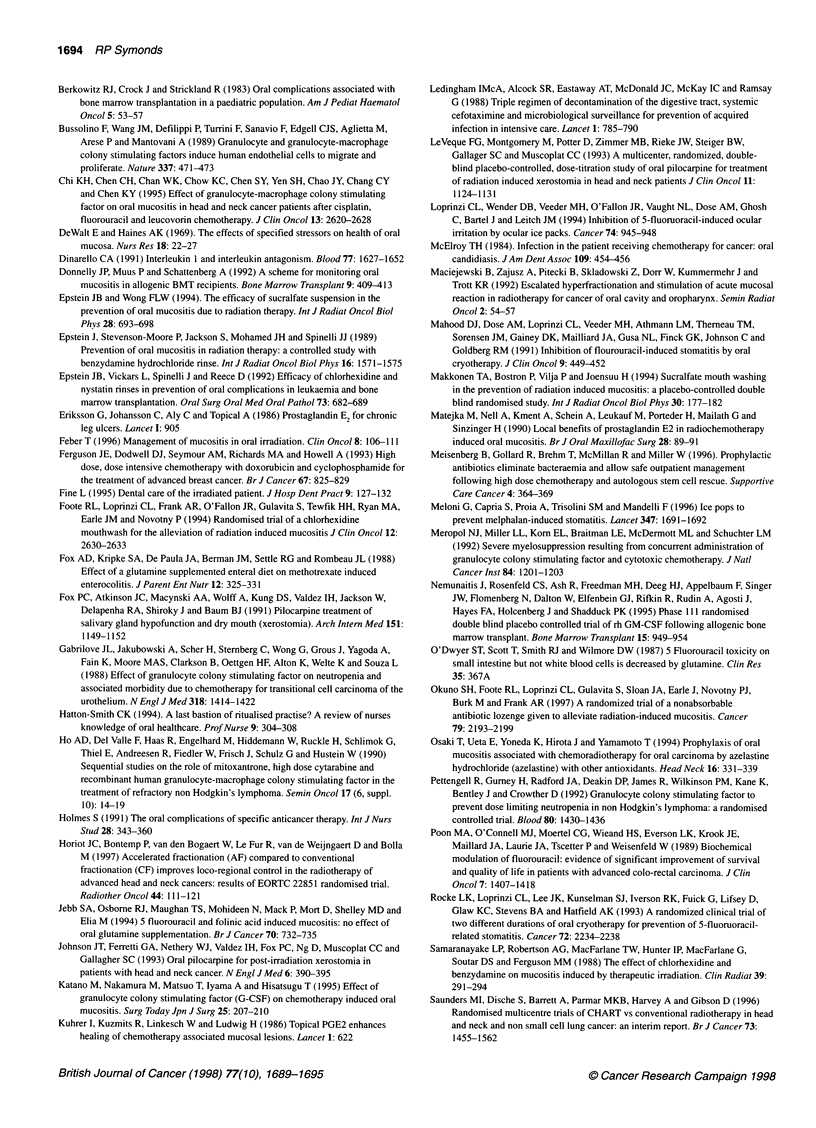

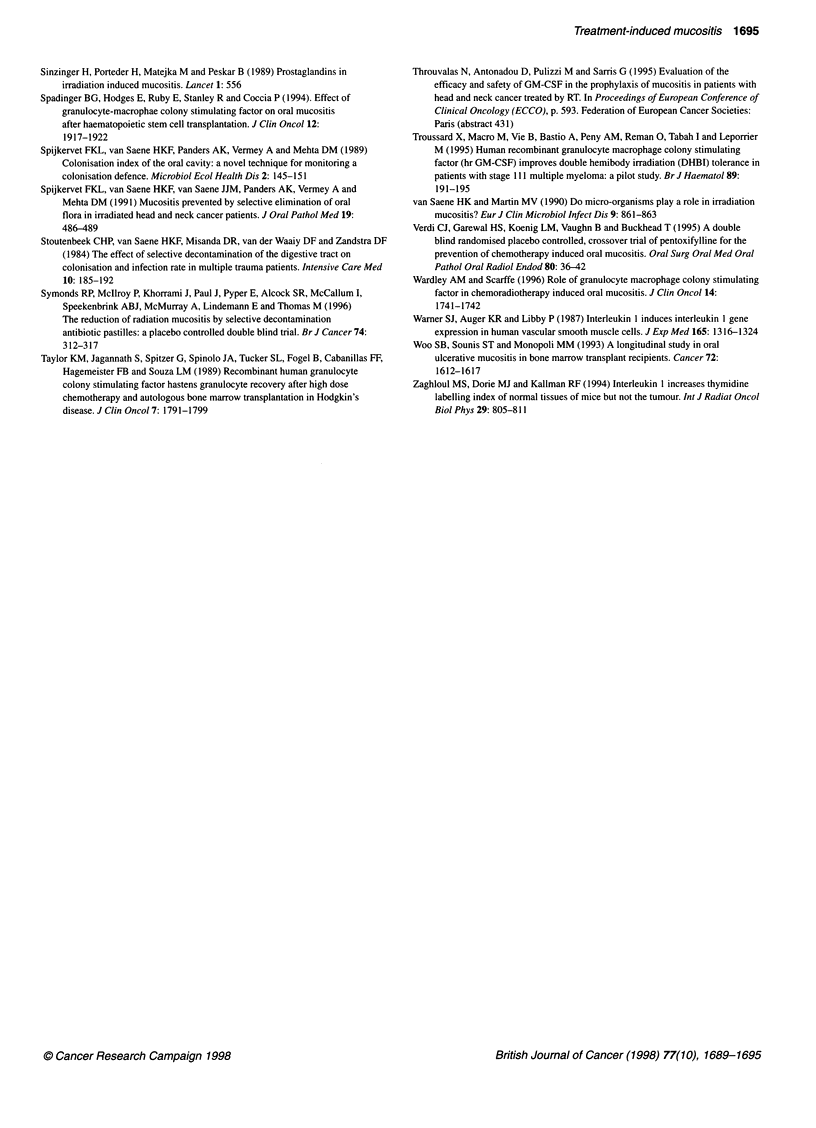


## References

[OCR_00781] Abouna G. M., Adnani M. S., Kumar M. S., Samhan S. A. (1986). Fate of transplanted kidneys with diabetic nephropathy.. Lancet.

[OCR_00626] Allison R. R., Vongtama V., Vaughan J., Shin K. H. (1995). Symptomatic acute mucositis can be minimized or prophylaxed by the combination of sucralfate and fluconazole.. Cancer Invest.

[OCR_00629] Amdur R. J., Parsons J. T., Mendenhall W. M., Million R. R., Cassisi N. J. (1989). Split-course versus continuous-course irradiation in the postoperative setting for squamous cell carcinoma of the head and neck.. Int J Radiat Oncol Biol Phys.

[OCR_00635] Atkinson K., Biggs J. C., Downs K., Juttner C., Bradstock K., Lowenthal R. M., Dale B., Szer J. (1991). GM-CSF after allogeneic bone marrow transplantation: accelerated recovery of neutrophils, monocytes and lymphocytes.. Aust N Z J Med.

[OCR_00642] Barker G., Loftus L., Cuddy P., Barker B. (1991). The effects of sucralfate suspension and diphenhydramine syrup plus kaolin-pectin on radiotherapy-induced mucositis.. Oral Surg Oral Med Oral Pathol.

[OCR_00647] Barkvoll P., Attramadal A. (1989). Effect of nystatin and chlorhexidine digluconate on Candida albicans.. Oral Surg Oral Med Oral Pathol.

[OCR_00650] Berger A., Henderson M., Nadoolman W., Duffy V., Cooper D., Saberski L., Bartoshuk L. (1995). Oral capsaicin provides temporary relief for oral mucositis pain secondary to chemotherapy/radiation therapy.. J Pain Symptom Manage.

[OCR_00661] Berkowitz R. J., Crock J., Strickland R., Gordon E. M., Strandjord S., Coccia P. F. (1983). Oral complications associated with bone marrow transplantation in a pediatric population.. Am J Pediatr Hematol Oncol.

[OCR_00666] Bussolino F., Wang J. M., Defilippi P., Turrini F., Sanavio F., Edgell C. J., Aglietta M., Arese P., Mantovani A. (1989). Granulocyte- and granulocyte-macrophage-colony stimulating factors induce human endothelial cells to migrate and proliferate.. Nature.

[OCR_00672] Chi K. H., Chen C. H., Chan W. K., Chow K. C., Chen S. Y., Yen S. H., Chao J. Y., Chang C. Y., Chen K. Y. (1995). Effect of granulocyte-macrophage colony-stimulating factor on oral mucositis in head and neck cancer patients after cisplatin, fluorouracil, and leucovorin chemotherapy.. J Clin Oncol.

[OCR_00678] DeWalt E. M., Haines A. K. (1969). The effects of specified stressors on healthy oral mucosa.. Nurs Res.

[OCR_00682] Dinarello C. A. (1991). Interleukin-1 and interleukin-1 antagonism.. Blood.

[OCR_00683] Donnelly J. P., Muus P., Schattenberg A., De Witte T., Horrevorts A., DePauw B. E. (1992). A scheme for daily monitoring of oral mucositis in allogeneic BMT recipients.. Bone Marrow Transplant.

[OCR_00691] Epstein J. B., Stevenson-Moore P., Jackson S., Mohamed J. H., Spinelli J. J. (1989). Prevention of oral mucositis in radiation therapy: a controlled study with benzydamine hydrochloride rinse.. Int J Radiat Oncol Biol Phys.

[OCR_00696] Epstein J. B., Vickars L., Spinelli J., Reece D. (1992). Efficacy of chlorhexidine and nystatin rinses in prevention of oral complications in leukemia and bone marrow transplantation.. Oral Surg Oral Med Oral Pathol.

[OCR_00686] Epstein J. B., Wong F. L. (1994). The efficacy of sucralfate suspension in the prevention of oral mucositis due to radiation therapy.. Int J Radiat Oncol Biol Phys.

[OCR_00701] Eriksson G., Johansson C., Aly A. (1986). Topical prostaglandin E2 for chronic leg ulcers.. Lancet.

[OCR_00705] Feber T. (1996). Management of mucositis in oral irradiation.. Clin Oncol (R Coll Radiol).

[OCR_00706] Ferguson J. E., Dodwell D. J., Seymour A. M., Richards M. A., Howell A. (1993). High dose, dose-intensive chemotherapy with doxorubicin and cyclophosphamide for the treatment of advanced breast cancer.. Br J Cancer.

[OCR_00713] Foote R. L., Loprinzi C. L., Frank A. R., O'Fallon J. R., Gulavita S., Tewfik H. H., Ryan M. A., Earle J. M., Novotny P. (1994). Randomized trial of a chlorhexidine mouthwash for alleviation of radiation-induced mucositis.. J Clin Oncol.

[OCR_00720] Fox A. D., Kripke S. A., De Paula J., Berman J. M., Settle R. G., Rombeau J. L. (1988). Effect of a glutamine-supplemented enteral diet on methotrexate-induced enterocolitis.. JPEN J Parenter Enteral Nutr.

[OCR_00725] Fox P. C., Atkinson J. C., Macynski A. A., Wolff A., Kung D. S., Valdez I. H., Jackson W., Delapenha R. A., Shiroky J., Baum B. J. (1991). Pilocarpine treatment of salivary gland hypofunction and dry mouth (xerostomia).. Arch Intern Med.

[OCR_00732] Gabrilove J. L., Jakubowski A., Scher H., Sternberg C., Wong G., Grous J., Yagoda A., Fain K., Moore M. A., Clarkson B. (1988). Effect of granulocyte colony-stimulating factor on neutropenia and associated morbidity due to chemotherapy for transitional-cell carcinoma of the urothelium.. N Engl J Med.

[OCR_00914] Gordon B., Spadinger A., Hodges E., Ruby E., Stanley R., Coccia P. (1994). Effect of granulocyte-macrophage colony-stimulating factor on oral mucositis after hematopoietic stem-cell transplantation.. J Clin Oncol.

[OCR_00740] Hatton-Smith C. K. (1994). A last bastion of ritualised practice? A review of nurses' knowledge of oral healthcare.. Prof Nurse.

[OCR_00744] Ho A. D., Del Valle F., Haas R., Engelhard M., Hiddemann W., Rückle H., Schlimok G., Thiel E., Andreesen R., Fiedler W. (1990). Sequential studies on the role of mitoxantrone, high-dose cytarabine, and recombinant human granulocyte-macrophage colony-stimulating factor in the treatment of refractory non-Hodgkin's lymphoma.. Semin Oncol.

[OCR_00753] Holmes S. (1991). The oral complications of specific anticancer therapy.. Int J Nurs Stud.

[OCR_00757] Horiot J. C., Bontemps P., van den Bogaert W., Le Fur R., van den Weijngaert D., Bolla M., Bernier J., Lusinchi A., Stuschke M., Lopez-Torrecilla J. (1997). Accelerated fractionation (AF) compared to conventional fractionation (CF) improves loco-regional control in the radiotherapy of advanced head and neck cancers: results of the EORTC 22851 randomized trial.. Radiother Oncol.

[OCR_00766] Jebb S. A., Osborne R. J., Maughan T. S., Mohideen N., Mack P., Mort D., Shelley M. D., Elia M. (1994). 5-fluorouracil and folinic acid-induced mucositis: no effect of oral glutamine supplementation.. Br J Cancer.

[OCR_00771] Johnson J. T., Ferretti G. A., Nethery W. J., Valdez I. H., Fox P. C., Ng D., Muscoplat C. C., Gallagher S. C. (1993). Oral pilocarpine for post-irradiation xerostomia in patients with head and neck cancer.. N Engl J Med.

[OCR_00791] LeVeque F. G., Montgomery M., Potter D., Zimmer M. B., Rieke J. W., Steiger B. W., Gallagher S. C., Muscoplat C. C. (1993). A multicenter, randomized, double-blind, placebo-controlled, dose-titration study of oral pilocarpine for treatment of radiation-induced xerostomia in head and neck cancer patients.. J Clin Oncol.

[OCR_00785] Ledingham I. M., Alcock S. R., Eastaway A. T., McDonald J. C., McKay I. C., Ramsay G. (1988). Triple regimen of selective decontamination of the digestive tract, systemic cefotaxime, and microbiological surveillance for prevention of acquired infection in intensive care.. Lancet.

[OCR_00799] Loprinzi C. L., Wender D. B., Veeder M. H., O'Fallon J. R., Vaught N. L., Dose A. M., Ghosh C., Bartel J., Leitch J. M. (1994). Inhibition of 5-fluorouracil-induced ocular irritation by ocular ice packs.. Cancer.

[OCR_00814] Mahood D. J., Dose A. M., Loprinzi C. L., Veeder M. H., Athmann L. M., Therneau T. M., Sorensen J. M., Gainey D. K., Mailliard J. A., Gusa N. L. (1991). Inhibition of fluorouracil-induced stomatitis by oral cryotherapy.. J Clin Oncol.

[OCR_00820] Makkonen T. A., Boström P., Vilja P., Joensuu H. (1994). Sucralfate mouth washing in the prevention of radiation-induced mucositis: a placebo-controlled double-blind randomized study.. Int J Radiat Oncol Biol Phys.

[OCR_00825] Matejka M., Nell A., Kment G., Schein A., Leukauf M., Porteder H., Mailath G., Sinzinger H. (1990). Local benefit of prostaglandin E2 in radiochemotherapy-induced oral mucositis.. Br J Oral Maxillofac Surg.

[OCR_00804] McElroy T. H. (1984). Infection in the patient receiving chemotherapy for cancer: oral considerations.. J Am Dent Assoc.

[OCR_00830] Meisenberg B., Gollard R., Brehm T., McMillan R., Miller W. (1996). Prophylactic antibiotics eliminate bacteremia and allow safe outpatient management following high-dose chemotherapy and autologous stem cell rescue.. Support Care Cancer.

[OCR_00837] Meloni G., Capria S., Proia A., Trisolini S. M., Mandelli F. (1996). Ice pops to prevent melphalan-induced stomatitis.. Lancet.

[OCR_00841] Meropol N. J., Miller L. L., Korn E. L., Braitman L. E., MacDermott M. L., Schuchter L. M. (1992). Severe myelosuppression resulting from concurrent administration of granulocyte colony-stimulating factor and cytotoxic chemotherapy.. J Natl Cancer Inst.

[OCR_00847] Nemunaitis J., Rosenfeld C. S., Ash R., Freedman M. H., Deeg H. J., Appelbaum F., Singer J. W., Flomenberg N., Dalton W., Elfenbein G. J. (1995). Phase III randomized, double-blind placebo-controlled trial of rhGM-CSF following allogeneic bone marrow transplantation.. Bone Marrow Transplant.

[OCR_00860] Okuno S. H., Foote R. L., Loprinzi C. L., Gulavita S., Sloan J. A., Earle J., Novotny P. J., Burk M., Frank A. R. (1997). A randomized trial of a nonabsorbable antibiotic lozenge given to alleviate radiation-induced mucositis.. Cancer.

[OCR_00867] Osaki T., Ueta E., Yoneda K., Hirota J., Yamamoto T. (1994). Prophylaxis of oral mucositis associated with chemoradiotherapy for oral carcinoma by Azelastine hydrochloride (Azelastine) with other antioxidants.. Head Neck.

[OCR_00872] Pettengell R., Gurney H., Radford J. A., Deakin D. P., James R., Wilkinson P. M., Kane K., Bentley J., Crowther D. (1992). Granulocyte colony-stimulating factor to prevent dose-limiting neutropenia in non-Hodgkin's lymphoma: a randomized controlled trial.. Blood.

[OCR_00879] Poon M. A., O'Connell M. J., Moertel C. G., Wieand H. S., Cullinan S. A., Everson L. K., Krook J. E., Mailliard J. A., Laurie J. A., Tschetter L. K. (1989). Biochemical modulation of fluorouracil: evidence of significant improvement of survival and quality of life in patients with advanced colorectal carcinoma.. J Clin Oncol.

[OCR_00887] Rocke L. K., Loprinzi C. L., Lee J. K., Kunselman S. J., Iverson R. K., Finck G., Lifsey D., Glaw K. C., Stevens B. A., Hatfield A. K. (1993). A randomized clinical trial of two different durations of oral cryotherapy for prevention of 5-fluorouracil-related stomatitis.. Cancer.

[OCR_00893] Samaranayake L. P., Robertson A. G., MacFarlane T. W., Hunter I. P., MacFarlane G., Soutar D. S., Ferguson M. M. (1988). The effect of chlorhexidine and benzydamine mouthwashes on mucositis induced by therapeutic irradiation.. Clin Radiol.

[OCR_00900] Saunders M. I., Dische S., Barrett A., Parmar M. K., Harvey A., Gibson D. (1996). Randomised multicentre trials of CHART vs conventional radiotherapy in head and neck and non-small-cell lung cancer: an interim report. CHART Steering Committee.. Br J Cancer.

[OCR_00910] Sinzinger H., Porteder H., Matejka M., Peskar B. A. (1989). Prostaglandins in irradiation-induced mucositis.. Lancet.

[OCR_00925] Spijkervet F. K., van Saene H. K., van Saene J. J., Panders A. K., Vermey A., Mehta D. M. (1990). Mucositis prevention by selective elimination of oral flora in irradiated head and neck cancer patients.. J Oral Pathol Med.

[OCR_00932] Stoutenbeek C. P., van Saene H. K., Miranda D. R., Zandstra D. F. (1984). The effect of selective decontamination of the digestive tract on colonisation and infection rate in multiple trauma patients.. Intensive Care Med.

[OCR_00939] Symonds R. P., McIlroy P., Khorrami J., Paul J., Pyper E., Alcock S. R., McCallum I., Speekenbrink A. B., McMurray A., Lindemann E. (1996). The reduction of radiation mucositis by selective decontamination antibiotic pastilles: a placebo-controlled double-blind trial.. Br J Cancer.

[OCR_00947] Taylor K. M., Jagannath S., Spitzer G., Spinolo J. A., Tucker S. L., Fogel B., Cabanillas F. F., Hagemeister F. B., Souza L. M. (1989). Recombinant human granulocyte colony-stimulating factor hastens granulocyte recovery after high-dose chemotherapy and autologous bone marrow transplantation in Hodgkin's disease.. J Clin Oncol.

[OCR_00962] Troussard X., Macro M., Vie B., Batho A., Peny A. M., Reman O., Tabah I., Leporrier M. (1995). Human recombinant granulocyte-macrophage colony stimulating factor (hrGM-CSF) improves double hemibody irradiation (DHBI) tolerance in patients with stage III multiple myeloma: a pilot study.. Br J Haematol.

[OCR_00974] Verdi C. J., Garewal H. S., Koenig L. M., Vaughn B., Burkhead T. (1995). A double-blind, randomized, placebo-controlled, crossover trial of pentoxifylline for the prevention of chemotherapy-induced oral mucositis.. Oral Surg Oral Med Oral Pathol Oral Radiol Endod.

[OCR_00981] Wardley A. M., Scarffe J. H. (1996). Role of granulocyte-macrophage colony-stimulating factor in chemotherapy-induced oral mucositis.. J Clin Oncol.

[OCR_00986] Warner S. J., Auger K. R., Libby P. (1987). Human interleukin 1 induces interleukin 1 gene expression in human vascular smooth muscle cells.. J Exp Med.

[OCR_00989] Woo S. B., Sonis S. T., Monopoli M. M., Sonis A. L. (1993). A longitudinal study of oral ulcerative mucositis in bone marrow transplant recipients.. Cancer.

[OCR_00994] Zaghloul M. S., Dorie M. J., Kallman R. F. (1994). Interleukin 1 increases thymidine labeling index of normal tissues of mice but not the tumor.. Int J Radiat Oncol Biol Phys.

[OCR_00970] van Saene H. K., Martin M. V. (1990). Do microorganisms play a role in irradiation mucositis?. Eur J Clin Microbiol Infect Dis.

